# Species-Specific Effects on Throughfall Kinetic Energy in Subtropical Forest Plantations Are Related to Leaf Traits and Tree Architecture

**DOI:** 10.1371/journal.pone.0128084

**Published:** 2015-06-16

**Authors:** Philipp Goebes, Helge Bruelheide, Werner Härdtle, Wenzel Kröber, Peter Kühn, Ying Li, Steffen Seitz, Goddert von Oheimb, Thomas Scholten

**Affiliations:** 1 University of Tübingen, Department of Geosciences, Institute of Geography, Chair of Soil Science and Geomorphology, Rümelinstraße 19–23, 72070, Tübingen, Germany; 2 Martin-Luther-University Halle Wittenberg, Geobotany, am Kirchtor 1, 06108, Halle, Germany; 3 German Centre for Integrative Biodiversity Research (iDiv) Halle-Jena-Leipzig, Deutscher Platz 5e, 04103, Leipzig, Germany; 4 Leuphana University Lüneburg, Faculty of Sustainability, Institute of Ecology, Scharnhorststraße 1, 21335, Lüneburg, Germany; 5 Technische Universität Dresden, Institute of General Ecology and Environmental Protection, Pienner Str. 7, 01737, Tharandt, Germany; DOE Pacific Northwest National Laboratory, UNITED STATES

## Abstract

Soil erosion is a key threat to many ecosystems, especially in subtropical China where high erosion rates occur. While the mechanisms that induce soil erosion on agricultural land are well understood, soil erosion processes in forests have rarely been studied. Throughfall kinetic energy (TKE) is influenced in manifold ways and often determined by the tree’s leaf and architectural traits. We investigated the role of species identity in mono-specific stands on TKE by asking to what extent TKE is species-specific and which leaf and architectural traits account for variation in TKE. We measured TKE of 11 different tree species planted in monocultures in a biodiversity-ecosystem-functioning experiment in subtropical China, using sand-filled splash cups during five natural rainfall events in summer 2013. In addition, 14 leaf and tree architectural traits were measured and linked to TKE. Our results showed that TKE was highly species-specific. Highest TKE was found below *Choerospondias axillaris* and *Sapindus saponaria*, while *Schima superba* showed lowest TKE. These species-specific effects were mediated by leaf habit, leaf area (LA), leaf pinnation, leaf margin, stem diameter at ground level (GD), crown base height (CBH), tree height, number of branches and leaf area index (LAI) as biotic factors and throughfall as abiotic factor. Among these, leaf habit, tree height and LA showed the highest effect sizes on TKE and can be considered as major drivers of TKE. TKE was positively influenced by LA, GD, CBH, tree height, LAI, and throughfall amount while it was negatively influenced by the number of branches. TKE was lower in evergreen, simple leaved and dentate leaved than in deciduous, pinnated or entire leaved species. Our results clearly showed that soil erosion in forest plantations can be mitigated by the appropriate choice of tree species.

## Introduction

Soil erosion negatively influences ecosystems widely, especially in regions with high erosion rates such as subtropical China [1]. Soil erosion brings about high economic costs due to declining agricultural productivity, reduced soil organic matter, relocation of nutrients, and off-site effects that influence human safety and food security [2–4]. Therefore, soil erosion plays an important ecological and economic role [5]. Reducing soil erosion is often achieved by afforestation [6], due to a high surface cover and stabilized soil aggregates in forests [7]. Afforestation in subtropical regions is dominated by mono-specific stands [8], primarily in order to optimize wood production in terms of quantity and quality by planting fast-growing species and to allow for a simple and standardized management [9]. Afforestations are well acknowledged for their great contribution in meeting the increasing demand for wood products and in carbon sequestration, thus having strong implications for climate change mitigation [10].

Soil erosion in forests is highly influenced by throughfall kinetic energy (TKE) [11]. TKE is a combination of the drop size distribution and drop velocity of throughfall. It is known that forests highly influence the kinetic energy of rainfall as first step towards erosion occurrence by their structure and species composition [5,12,13]. Many studies on throughfall have been conducted [14,15], but there can be different mechanisms if TKE is examined. Even though soil erosion is generally reduced in forests [7], TKE can be higher in forests than in open fields [16,17]. In particular, with a sparse understory vegetation and leaf litter cover TKE can strongly increase soil erosion under forest.

In open field sites, kinetic energy of rainfall is only affected by abiotic factors (i.e., rainfall intensity and amount; [18,19]. Below forest canopies, however, biotic factors come into play with the potential to alter throughfall and TKE considerably. As a result, large species-specific differences have been found [16,20–23]. Species-specific effects on TKE are evoked by plant traits such as leaf area index [13,21,24], leaf habit [5], tree height [12], canopy thickness [18,25], branch characteristics [26] and the first branch of a tree individual [5,25]. The mechanism of the latter is that rain is channeled by leaves and branches to drop at specific spatial points above the ground surface resulting in smaller or larger rain drops [26]. Hence, this is one of the reasons for an increased TKE, and thus soil erosion potential, on spatially confined soil patches at the micro scale. As an example of species-specific differences evoked by leaf traits, species with broad leaves and a rough cuticle produced larger drops than species with smaller and wax-coated leaves and might, thus, increase TKE [27]. For this reason, TKE under the canopy of *Schima superba* with evergreen leaves was found to be lower than under that of *Castanea henryi* and *Quercus serrata* with deciduous leaves [5]. However, most preceding studies have only dealt with at maximum four different species [5,15,16], precluding cross-species comparisons of TKE-trait relationships. One exception is the study of [27], who investigated nine different species with regard to their leaf drips but without considering plant traits. However, several studies have disregarded both the mediating effects of most biotic [28–31] and abiotic factors such as rainfall intensity and throughfall amount on TKE [18,19].

As a consequence, little is known about how and to what extent species-specific leaf and tree architectural traits mediate soil erosion processes under tree canopies. This in turn means that a broader set of species (covering a wide range of leaf and tree architectural traits) needs to be analyzed to reveal species identity effects on TKE. Therefore, it is essential to study TKE under a multitude of species that vary in leaf traits and morphology. Moreover, the investigation of several traits allows the identification of the major drivers for variations in TKE, independent of species identity. Major drivers for variations in TKE have been identified in intraspecific comparisons [32] and could be tested for their interspecific validity. However, literature reporting on TKE distribution under forest canopies remains scarce (both generally and in subtropical regions), underlining the need to further investigate TKE variation below broad-leaved tree species.

We set out to close this knowledge gap by quantifying relationships between TKE and leaf and tree architectural traits of 11 different tree species typical of subtropical broad-leaved forest ecosystems of China. Trees were grown in monocultures that were established in the context of a large-scale biodiversity-ecosystem functioning experiment (henceforth referred to as BEF-China; [33]). Specifically, we tested the following hypotheses:

H1: TKE below forest canopies is highly species-specific.

H2: Leaf and tree architectural traits mediate species-specific effects on TKE.

## Materials and Methods

### Study site

The BEF-China experiment is located near Xingangshan Township, Jiangxi Province (N29°08–11, E117°90–93), P.R. China. The mean annual temperature is 17.4°C and mean annual rainfall is 1635 mm. The climate of the study area is characterized by subtropical summer monsoon with a wet season from May to July and a dry winter. After the clear-cut of a *Cunninghamia lanceolata* plantation in 2008, an experimental forest was planted on a plot-level based approach with 400 tree individuals per plot (25.8 m x 25.8 m; planted in 20 rows of 20 tree individuals each), using a planting distance of 1.29 m and including a total of 24 tree species on 261 plots to investigate biodiversity effects on ecosystem functions (see [33] and [34] for detailed explanations). This study focuses only on monoculture plots of trees that ranged in mean height from 1.10 m to 5.76 m in 2013. At the time of study, the trees were five years old. No specific permissions were required for these locations and activities. The field studies did not involve endangered or protected species.

### Experimental design and data sampling

TKE was measured during five rainfall events with an event-based approach for a total of 11 species in 17 monoculture plots in 2013. Within the central part of each plot (including 6 x 6 trees), eight randomly assigned positions with distinct distances to the tree stems were used to measure TKE (1) 15 cm away from tree stem, (2) in the middle of two tree individuals, (3) in the middle of four individuals, (4) 45 cm away from tree stem, (5) at the 45 cm x 120 cm intersection between two individuals, (6) below the first branch of a tree individual, (7) at the 75 cm x 75 cm intersection between two individuals, and (8) 30 cm away from tree stem). TKE was measured using splash cups [35] and representative values of J/m² were obtained by using a modified version of the function provided by [35]. Next to each splash cup, a rainfall collector was installed to quantify throughfall with a high spatial resolution. Rainfall events were registered by the BEF-China climate stations and classified by rainfall intensity, duration and total amount (S3 Table). A total of eight leaf traits and six architectural traits were analyzed. Leaf traits included leaf area (LA), specific leaf area (SLA), leaf pinnation (simple or pinnate), leaf margin (entire or dentate), trichome cover of upper leaf surface, leaf thickness, leaf toughness and leaf habit (deciduous or evergreen). These traits were measured on individuals planted in the experiment [36,37]. Architectural traits examined at each tree individual were total height, elliptic crown area, number of branches, stem diameter at ground level (GD) and crown base height (CBH) [38]. Leaf area index (LAI) was registered at each TKE measuring point under diffuse radiation conditions, using a Nikon D100 with a Nikon AF G DX 180° and HemiView V8 (Delta-T) [39]. Table 1 gives an overview of all tree species with leaf and architectural traits influencing TKE.

**Table 1:** Leaf and tree architectural traits of the tree species included in the present study according to a significant influence on throughfall kinetic energy.

Species name	Abbrev. of species name	Leaf area index (LAI)	Leaf area (LA) [mm²]	Leaf habit	Leaf pinnation	Leaf margin	Tree height [m]	Number of branches	Crown base height (CBH) [m]	Mean Through-fall [mm]
*Castanea henryi* Rhed. & Wils.	cah	2.77	3,128	D	S	D	4.88	19	0.82	55.7
*Choerospondias axillaris* (Roxb.) Burtt & Hill	cha	2.31	35,484	D	P	D	5.76	12	2.69	74.6
*Cyclobalanopsis glauca* (Thunb.) Oerst.	cyg	0.29	2,474	E	S	D	1.43	16	0.29	66.7
*Koelreuteria bipinnata* Franch.	kob	0.27	30,727	D	P	D	1.19	1	0.59	78.3
*Liquidambar formosana* Hance	lif	1.06	5,051	D	S	D	2.25	32	0.24	69.8
*Lithocarpus glaber* (Thunb.) Nakai	lig	0.77	1,956	E	S	E	1.92	27	0.30	50.4
*Quercus fabri* Hance	quf	0.55	1,912	D	S	D	1.66	24	0.36	65.7
*Quercus serrata* Murray	qus	0.41	1,972	D	S	D	1.10	23	0.16	78.2
*Sapindus saponaria* Linn.	sas	1.13	42,231	D	S	E	2.33	5	0.68	75.1
*Triadica sebifera* Small	trs	1.20	2,108	D	S	E	2.65	19	0.33	70.5
*Schima superba* Garder & Champion	scs	3.06	3,230	E	S	D	3.38	47	0.42	39.8

Values represent means of the variables measured. Abbreviations: D = deciduous, E = evergreen, S = simple, P = pinnate, D = dentate, E = entire. Mean throughfall refers to the mean across all rainfall events.

### Data analyses

Species-specific variation of TKE was investigated using linear mixed-effect models fitted by restricted maximum likelihood. Rainfall event, species identity and the interaction of species identity with rainfall event were included as fixed factors. The interaction of species identity with rainfall event allowed detecting whether differences among species only played a role at certain rainfall events. Plot, measurement position within each plot, interaction of plot with rainfall event and interaction of plot with position entered the model as random effects. For testing effects within each rainfall event, rainfall event was not used as fixed factor. Contrasts were fitted before species identity to detect species which had significantly higher or lower TKE than mean of all others. Significant effects were detected using Wald Test statistics with Type I SS ANOVAs. In total, 625 data points entered the analyses (5 events x 17 plots x 8 positions– 55 failed measurements).

To specify possible effects of species identity, mediation analysis were constructed by fitting mediation trait variables before the species identity term. Mediation variables were detected as such, if significance of species identity was changed from significance to non-significance and if the mediation variable itself significantly influenced TKE. To identify the most important mediation variable, categorical levels were predicted and ranked by their magnitude of TKE differences (effect size). For continuous mediation variables, the difference in TKE was evaluated when increasing mediation variable by one standard deviation. Each model was only fitted with a single mediation variable to avoid multicollinearity among traits and overparameterization.

Additionally, a model was constructed consisting only of leaf traits (fitted first to avoid underrepresentation by larger effects), tree architectural traits and throughfall to test for their influences on TKE. In this model, plot and rainfall event were considered as random effects. Model simplification was done using step-wise backward selection with the maximum likelihood approach [40]. Hence, the final model only contained significant effects (P<0.05). Prior to the analyses, all covariates have been checked for collinearity (correlations were not allowed to exceed R = ± 0.7). Hence, leaf toughness, leaf thickness and crown area were omitted in the final model due to multicollinearity. Predictions were used to identify the effect size according to the method described above.

If a measuring position was influenced by more than one tree individuals, mean values of leaf and tree architectural traits of surrounding individuals have been calculated.

TKE data was log-transformed to ensure normal distribution. Model residuals did not show violation of model assumptions (normality and homogeneity of variances). Analyses were conducted using R 2.14.1 [41]. Linear mixed effects models were analyzed with R package “asreml” [42] and “lme4” [43].

## Results

Across all rainfall events, species and plots, TKE was highly variable ranging from 7 J/m² to 2882 J/m². Mean TKE was 494 ± 536 J/m² and differences between rainfall events were considerable. Rainfall event 4 and 5 yielded the lowest (74 ± 54 J/m²) and the highest mean TKE (1247 ± 617 J/m²), respectively. In all models, TKE was strongly positively correlated with rainfall event (*F*_*4*,22_ = 731, *P* < 0.001).

**Fig 1:**
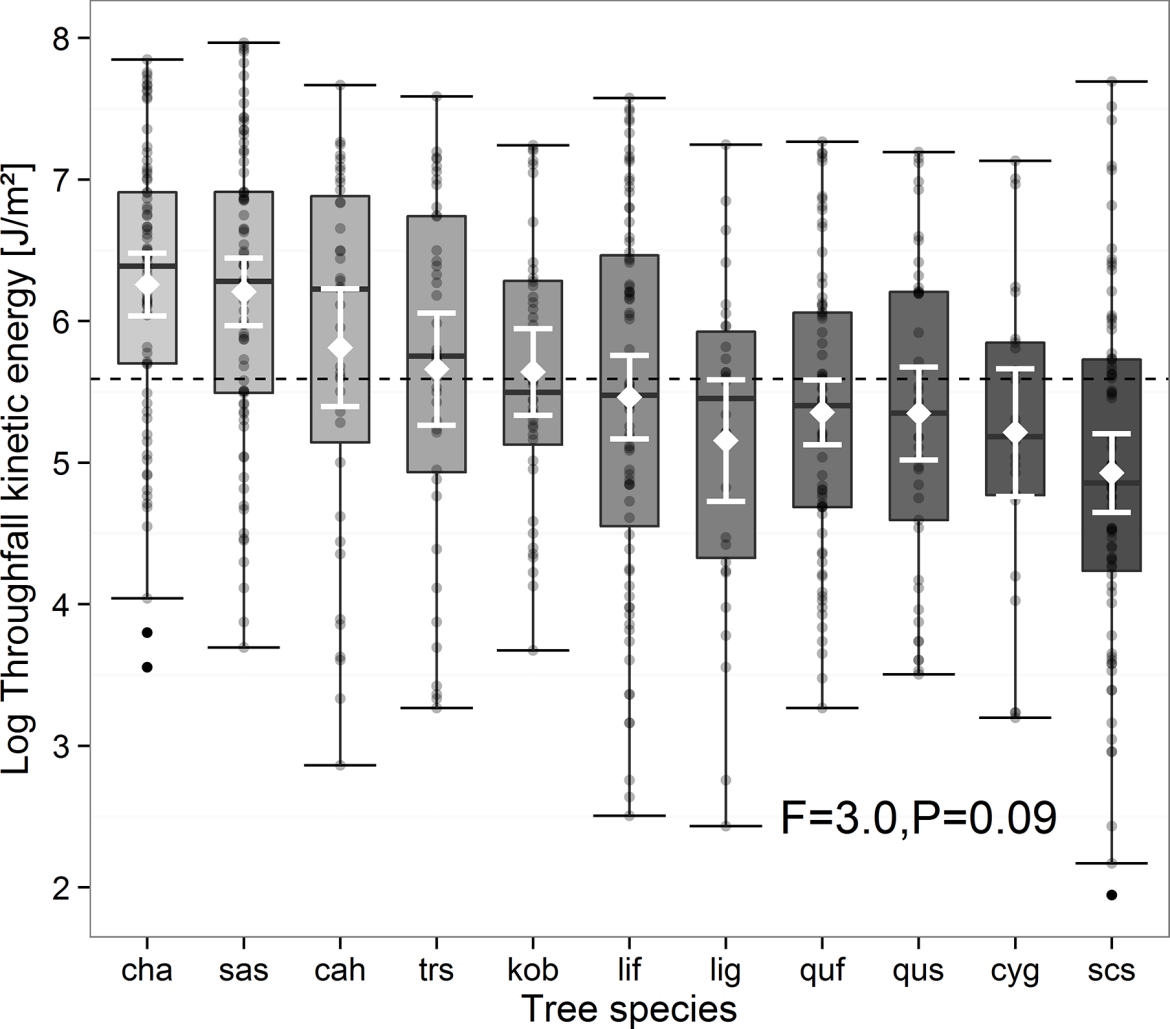
Throughfall kinetic energy (TKE, log-transformed) of the 11 species analyzed. Dotted line represents the total mean TKE. Inside the boxplots white rectangles represent mean and white bars standard deviation. For abbreviations of species names see Table 1.

TKE was species-specific but independent of a specific rainfall event. However, the species-specific effect size strongly depended on the rainfall event (Fig 1, S1 Table). Species identity significantly affected TKE at rainfall events 1 and 2 (*F*_1,6_ = 6.3, *P* < 0.05 and *F*_1,6_ = 4.6, *P* < 0.05, respectively), whereas it was not significantly related to TKE at rainfall events 3, 4, and 5. TKE below the canopy of *Choerospondias axillaris* and *Sapindus saponaria* were significantly higher (58%, *F*_1,6_ = 11.89, *P* = 0.013, and 62%, *F*_1,6_ = 10.11, *P* = 0.019, respectively) and TKE below the canopy of *Schima superba* was significantly lower (42%, *F*_1,6_ = 8.63, *P* = 0.026) than the mean TKE of all other species.

The effect of species identity on TKE was mediated by leaf habit, leaf pinnation, LA, tree height, LAI, SLA, throughfall, CBH, GD, crown area and number of branches (Table 2). Regarding categorical traits, the highest difference between factor levels occurred between different leaf habits (with a 92% increase of mean TKE from evergreen to deciduous). Increase of mean TKE for pinnated leaves was 60%. Considering vegetation continuous traits, high effect sizes were found for LA (+ 92%), tree height (+ 33%), LAI (- 25%), SLA (+ 17%), throughfall and CBH (each + 16%). Effect sizes were small for GD and the number of branches (all < 7%).

**Table 2:** Effect sizes of mediation variables (leaf and tree architectural traits).

		Change in TKE [J/m²] by changing mediation variable by one SD
Mediation variables	Leaf area	+ 199 **
	Leaf habit	+ 146 **
	Leaf pinnation	+ 141 *
	Height	+ 91 ***
	Leaf area index	- 65 **
	Crown base height	+ 46 **
	Throughfall amount	+ 42 ***
	Ground diameter	+ 16
	Number of branches	- 13 **

Values are predicted from mixed effect models for throughfall kinetic energy (TKE) with basic design structure (not shown, see S1 Table). For abbreviations of traits see Table 1.

***p < 0.001

**p < 0.01

*p < 0.05;. p < 0.1.

**Fig 2:**
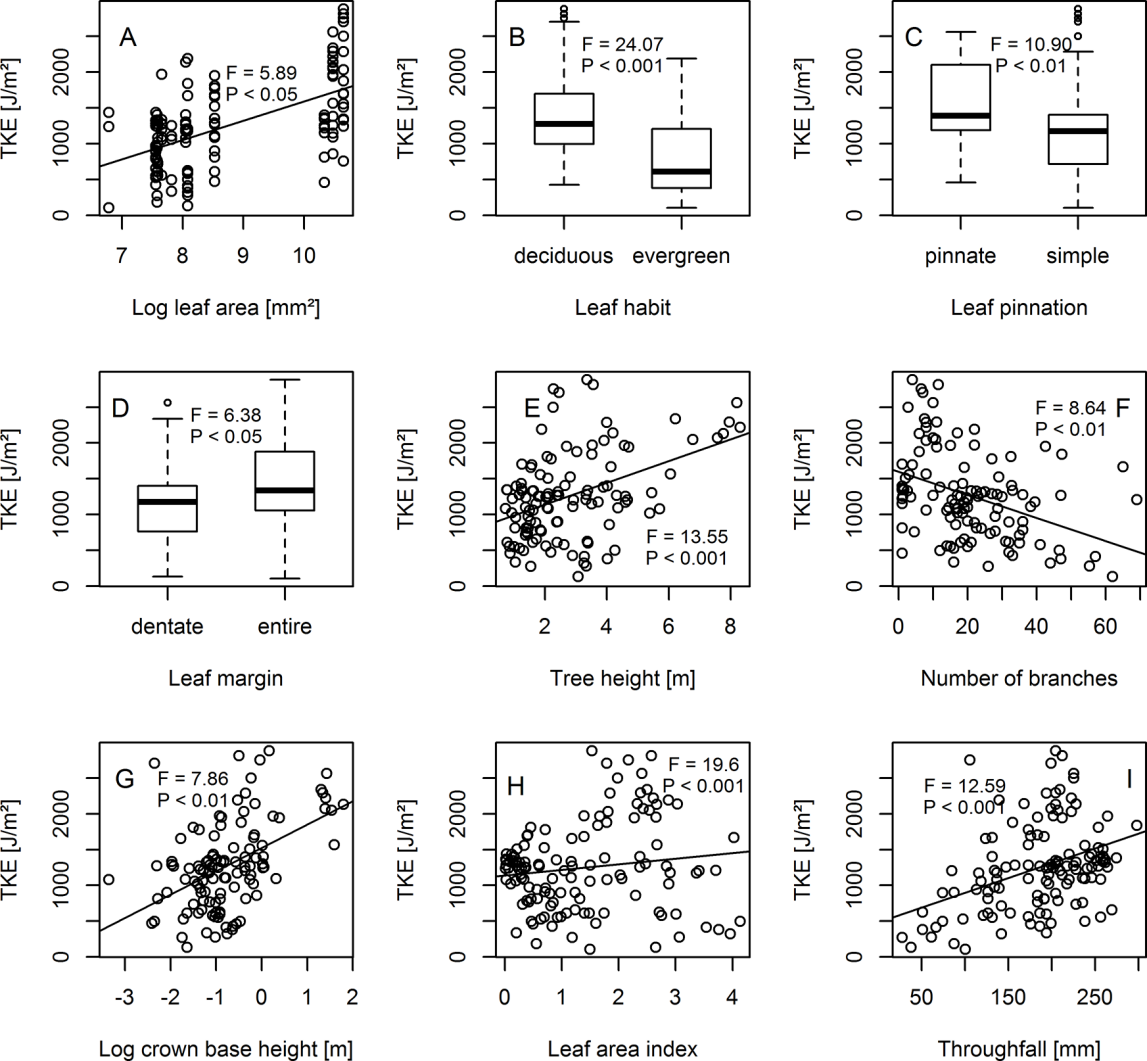
Throughfall kinetic energy (TKE) versus leaf traits (A-E), tree architectural traits (F-H) and abiotic covariates (I). Black solid lines indicate linear trend.

In general, TKE was significantly positively related to LA, CBH, height, and throughfall but negatively influenced by LAI and the number of branches. Moreover, deciduous species (+ 13 J/m²), species with pinnate (+ 32 J/m²) and entire margined (20 J/m²) leaves displayed higher TKE than evergreen species, species with simple leaves and species with dentate leave margins, respectively (Fig 2, S2 Table).

## Discussion

This study compared TKE of 11 tree species in monocultures typical of subtropical forest ecosystems in China. Further, it complemented former analyses by deepening our understanding of leaf traits and tree architecture effects on TKE [5,27]. Mediation analyses linked leaf and tree architectural traits to varying TKE induced by different tree species, and major biotic drivers of TKE variability were detected by comparing effect sizes. Finally, this study aimed to support the selection of appropriate tree species for tree plantation in order to minimize TKE and thus to counteract soil erosion in subtropical regions resulting from high monsoon precipitation (particularly in areas with steep terrain).

### Species-specific TKE variation (H1)

Our first hypothesis was confirmed by the significant influence of species identity on TKE. However, only three out of 11 species showed distinct differences in TKE compared to overall means. Among these, two species positively (*Choerospondias axillaris* and *Sapindus saponaria*) and one species negatively (*Schima superba*) affected TKE (Fig 1). Species-specific differences of throughfall amount or interception have been frequently reported [18,44]. Moreover, drop size distribution as an important driver of TKE has been found to be species-specific [27]. Nevertheless, preceding studies found no significant difference in TKE among certain species [5,16,21,44,45], which is in line with our findings. Furthermore, the highly significant interaction of species identity with rainfall event emphasizes the importance of abiotic characteristics in TKE distribution [18]. An influence of species identity on TKE was found at low-peak intensity rainfall events, whereas TKE at high rainfall intensities was not species-specific. An exception occurred at rainfall event 4 where species identity did not affect TKE although rainfall intensity was low. Higher intensity rainfall usually results in considerable canopy vibration, through which the drop sizes are reduced [20]. Therefore, the variation of TKE at high intensity rainfall could be much less than that at low intensity rainfall leading to no species-specific differences. However, this effect often is superimposed by an increase of total throughfall amount with higher rainfall intensities.

The species-specific effects of canopies of *Choerospondias axillaris*, *Sapindus saponaria* and *Schima superba* have strong implication for managing TKE. Planting Schima superba, which negatively affected TKE, has the potential to decrease soil erosion in early successional stages. *Schima superba* is also well-known for high values of canopy interception during rainfall [46]. This could be partially attributed to the high LAI and re-interception of rainfall by lower canopy layers [25,30]. Low TKE below *Schima superba* was also reported by [5]. These findings are as much more relevant as *Schima superba* represents one of the dominant tree species in the regional species pool [47,48]. *Choerospondias axillaris* increased TKE which is consistent with high runoff volumes found for this species in comparison to peanut crops [49]. However, despite a TKE increase, higher soil loss with *Choerospondias axillaris* can be counteracted by an intact litter cover [29].

### Leaf and tree architectural traits mediate species-specific variation (H2)

First, the strong impact of rainfall event on TKE suggests that the TKE variation is pre-determined by the characteristics of rainfall events, such as duration, total rain amount, wind speed and rainfall intensity [18]. However, our study could not confirm a significant effect of rainfall duration and wind speed on TKE. Within a specific rainfall event, TKE differed among tree species as species responded differently to different rainfall intensities [50]. Independent of a specific rainfall event, the species identity effects on TKE were mediated by leaf and tree architectural traits. LA, leaf habit, leaf pinnation, GD, CBH, tree height, number of branches and LAI as biotic factors were found to be responsible in mediating species-specific TKE (Table 2). Moreover, the significant effect of throughfall measured at each splash cup position on TKE showed the influence of biotic and abiotic factors on TKE [18].

In our study, species-specific changes of TKE were induced most by leaf area. A higher leaf area can increase the gathering of rain water and thus may cause larger drops resulting in higher TKE [13,20,26]. In contrast, many studies have reported on the positive influence of leaf area on interception [44], which leads to decreasing throughfall amount and decreasing TKE. Therefore, in our study variation of interception might only play a minor role in explaining species-specific differences in TKE, since all rain events lasted long enough to compensate the effect of canopy storage at the beginning of each event. However, the high effect size of LA in our study might be an overestimation, since leaf areas of *Choerospondias axillaris* and *Sapindus saponaria*, both with largest TKE, were almost twice of the standard deviation above the mean. This is due to the fact that for measurements of leaf area, the leaflets of pinnate leaves are traditionally added up to a total value per pinnate leaf [51]. Furthermore, water might gather at the branch, where each leaflet splits, which in turn may result in increased drop size and thus TKE.

TKE varied second-most between deciduous and evergreen species where deciduous species showed higher TKE. Similarly, [5] found that *Castanea henryi* and *Quercus serrata* as examples of deciduous species yielded higher TKE than the evergreen species *Schima superba*. Leaf habit represents a dominant segregation for many leaf traits and has been found to influence core functional and physiological processes specifically in the study species [47] as well as globally [52–54]. Deciduous species tend to have leaves with higher SLA [55], which we found to positively affect TKE. In addition, evergreen species tended to have a larger crown length ratio (ratio of crown length to the total tree height). Two mechanisms might elucidate the great variation between deciduous and evergreen species: (i) A lower tree height decreases falling height of raindrops and thus, results in lower TKE; (ii) a larger crown area with lower tree height (higher crown length ration) may increase LAI which results in higher interception, leading to decreasing throughfall. Moreover, leaf pinnation (pinnate or simple) can alter drop sizes. On the basis of higher margin circumference in relation to total leaf area [56], pinnate leaves create more dripping points. In addition, pinnate leaves showed the highest leaf area (see Table 1) with the exception of *Sapindus saponaria*. Corresponding to the above, a higher leaf area increases TKE. However, different leaf margins contributed only marginally to species-specific changes in TKE. As demonstrated in former studies [13,28], tree height was the most important tree architectural parameter to describe species-specific differences in TKE. Increasing tree height can contribute to higher TKE by several processes: (i) higher drop velocity due to higher falling heights [57], (ii) larger crown width [38] that increases drop size through increased confluence, and (iii) larger crown width is associated with higher LAI, which creates more dripping points [26,28]. The species-specific differences in TKE were mediated by LAI which negatively affected TKE. It is known that high canopy thickness increases drop splitting by dripping on branches and leaves [20,25], which in turn may decrease raindrop sizes. Moreover, higher canopy thickness in young forest stands might decrease space between vegetation and surface resulting in lower rain drop velocities [5]. Additionally, with denser and thicker crown cover water storage in the canopy increases, but this effect can be neglected with regard to rainfall durations longer than a day.

CBH (with half of the effect size of tree height) contributed to species variances as indirect factor, as it is usually related to tree height. CBH may constitute “the last barrier” in releasing throughfall drops, determines the falling height and thus drop velocity. Yet, our data showed that CBH contributed to interspecific TKE much less as compared to tree height. One reason might be that the same CBH might occur at trees along a large range of tree height.

GD positively and branch number negatively mediated species identity of TKE, but only to a smaller extent (< 7% difference). This suggests that GD mediated species-specific changes as an indirect effect of tree growth characteristics (tree height and LAI). Furthermore, the number of branches might affect TKE through an indirect effect via LAI. Branches gather throughfall and release it at any random position or transfer it directly to the stem, which decreases throughfall and increases stemflow [26]. However, our results indicated that this was a weak effect and water might both, be distributed along the branches and transferred to the stem in equal proportions [26]. Moreover, the greater effect size of LAI demonstrated that leaves are much more important than the branches as regards the impacts of species-specific TKE.

Besides the significant effect of plant traits on TKE, throughfall amount was also highly correlated with TKE, but showed smaller effect sizes than the findings from other studies [19]. In most studies, throughfall amount was found to be the major driver of spatial variability of TKE [16,35]. However, our species comparison revealed that shifts in drop formation and drop velocity within a specific rainfall event might have a higher impact on TKE than the total amounts of rainfall. Thus higher throughfall amounts do not necessarily lead to higher TKE at rainfall event level.

## Conclusion

This study aimed to contribute to a better understanding of mechanisms underlying the relationships between TKE and leaf and tree architectural traits, taking 11 tree species of subtropical forests in China as example. In conclusion, the optimal trait combination a tree should have to minimize TKE would be a low leaf area index and leaf area, simple pinnated leaves, dentated leaf margins, low tree height, high number of branches and a low crown base height. Furthermore, evergreen species showed lower TKE than deciduous ones. However, traits such as tree height, stem diameter and LAI will change with growing tree individuals while other traits such as SLA and all binary leaf morphological traits are believed not to change drastically during tree growth. These implications need to be considered when transferring our results to other systems with a fully developed crown cover.

Our results showed that TKE distribution among different species is much more complex than throughfall distribution solely. TKE sensitively responded to the amount of throughfall, but also to the transformation of throughfall amount (in terms of drop size and drop velocity) by leaf and tree architectural traits (Fig 2). Thus, this study helps to understand the interaction between these vegetation characteristics, species identity and TKE as a basis for erosion modeling and the mitigation of soil erosion by means of an optimized selection of appropriate tree species in the context of afforestation programs.

## Supporting Information

S1 Table Results from the basic mixed-effects model for throughfall kinetic energy response. (DOCX)

S2 Table Effect sizes of leaf and tree architectural traits. Values are predicted from the full multivariate mixed effect models for throughfall kinetic energy (TKE) with basic design structure (not shown, see S1 Table). For abbreviations of traits see Table 1.
(DOCX)

S3 Table Characteristics of the five rainfall events. (DOCX)
