# Supplementary material for: Species-Specific Effects on Throughfall Kinetic Energy in Subtropical Forest Plantations Are Related to Leaf Traits and Tree Architecture
Source: PLoS One. 2015 Jun 16;10(6):e0128084. doi: 10.1371/journal.pone.0128084 (PMC4469422; doi:10.1371/journal.pone.0128084)
Supplement: S1 Table — (DOCX) [file pone.0128084.s001.docx]

**Supporting Information**

|  |  | Df | DenDF | F | P |
| --- | --- | --- | --- | --- | --- |
| Fixed Effects | Intercept | 1 | 6.2 | 17760 | 1.2*10^-11^ *** |
|  | Event | 4 | 22.4 | 731.4 | 2.2*10^-16^ *** |
|  | Position | 7 | 101.3 | 1.3 | 0.2510 |
|  | Species identity | 11 | 6.3 | 3.0 | 0.0936 . |
|  | Species identity:event | 44 | 21.5 | 3.0 | 0.0037 ** |
|  |  |  |  |  |  |
|  |  | Gamma | Component | Std.error |  |
| Random effects | Plot | 0.4120 | 0.0797 | 0.0588 |  |
|  | Plot:Event | 0.0013 | 0.0003 | 0.0081 |  |
|  | Plot:Position | 0.6095 | 0.1180 | 0.0224 |  |
