# Supplementary material for: Species-Specific Effects on Throughfall Kinetic Energy in Subtropical Forest Plantations Are Related to Leaf Traits and Tree Architecture
Source: PLoS One. 2015 Jun 16;10(6):e0128084. doi: 10.1371/journal.pone.0128084 (PMC4469422; doi:10.1371/journal.pone.0128084)
Supplement: S2 Table — Values are predicted from the full multivariate mixed effect models for throughfall kinetic energy (TKE) with basic design structure (not shown, see S1 Table). For abbreviations of traits see Table 1. (DOCX) [file pone.0128084.s002.docx]

**Supporting Information**

|  | | Change in TKE [J/m²] by changing mediation variable by one SD |
| --- | --- | --- |
| Full model | Height | + 114 *** |
|  | LA | + 34 * |
|  | Leaf pinnation | + 31 ** |
|  | LAI | - 24 *** |
|  | Throughfall amount | + 21 *** |
|  | Leaf margin | + 20 * |
|  | Leaf habit | + 13 *** |
|  | Branch number | - 12 ** |
|  | CBH | + 3 ** |

***p < 0.001; **p < 0.01; *p < 0.05.
