# Supplementary material for: Species-Specific Effects on Throughfall Kinetic Energy in Subtropical Forest Plantations Are Related to Leaf Traits and Tree Architecture
Source: PLoS One. 2015 Jun 16;10(6):e0128084. doi: 10.1371/journal.pone.0128084 (PMC4469422; doi:10.1371/journal.pone.0128084)
Supplement: S3 Table — (DOCX) [file pone.0128084.s003.docx]

**Supporting Information**

| Rainfall events | Rainfall amount (mm) | Rainfall 5-min peak intensity (mm/h) | Rainfall intensity of total event (mm/h) |
| --- | --- | --- | --- |
| Event 1 | 23.3 | 12.1 | 2.29 |
| Event 2 | 39.3 | 22.8 | 3.42 |
| Event 3 | 61.2 | 44.4 | 4.25 |
| Event 4 | 6.6 | 25.2 | 2.83 |
| Event 5 | 185.7 | 127.2 | 6.07 |
